# A systematic review on papers that study on Single Nucleotide Polymorphism that affects coronavirus 2019 severity

**DOI:** 10.1186/s12879-022-07034-w

**Published:** 2022-01-12

**Authors:** Siyeon Suh, Sol Lee, Ho Gym, Sanghyuk Yoon, Seunghwan Park, Jihi Cha, Do-Hyung Kwon, YunSu Yang, Sun Ha Jee

**Affiliations:** 1 BASGENBIO Inc. LTD, Seoul, South Korea; 2 School of Biomedical Sciences, Li Ka Shing Faculty of Medicine, The University of Hong Kong, Pokfulam, Hong Kong; 3 Department of Epidemiology and Health Promotion, Institute for Health Promotion, Graduate School of Public Health, Yonsei University, 50-1 Yonsei-ro, Seodaemun-gu, Seoul, 03722 South Korea

## Abstract

**Background:**

COVID-19, caused by SARS-CoV-2 has become the most threatening issue to all populations around the world. It is, directly and indirectly, affecting all of us and thus, is an emerging topic dealt in global health. To avoid the infection, various studies have been done and are still ongoing. COVID-19 cases are reported all over the globe, and among the millions of cases, genetic similarity may be seen. The genetical common features seen within confirmed cases may help outline the tendency of infection and degree severity of the disease. Here, we reviewed multiple papers on SNPs related to SARS-CoV-2 infection and analyzed their results.

**Methods:**

The PubMed databases were searched for papers discussing SNPs associated with SARS-CoV-2 infection and severity. Clinical studies with human patients and statistically showing the relevance of the SNP with virus infection were included. Quality Assessment of all papers was done with Newcastle Ottawa Scale.

**Results:**

In the analysis, 21 full-text literature out of 2956 screened titles and abstracts, including 63,496 cases, were included. All were human-based clinical studies, some based on certain regions gathered patient data and some based on big databases obtained online. *ACE2, TMPRSS2,* and *IFITM3* are the genes mentioned most frequently that are related to SARS-CoV-2 infection. 20 out of 21 studies mentioned one or more of those genes. The relevant genes according to SNPs were also analyzed. rs12252-C, rs143936283, rs2285666, rs41303171, and rs35803318 are the SNPs that were mentioned at least twice in two different studies.

**Conclusions:**

We found that *ACE2, TMPRSS2,* and *IFITM3* are the major genes that are involved in SARS-CoV-2 infection. The mentioned SNPs were all related to one or more of the above-mentioned genes. There were discussions on certain SNPs that increased the infection and severity to certain groups more than the others. However, as there is limited follow-up and data due to a shortage of time history of the disease, studies may be limited.

## Introduction and background

In 2020, coronavirus disease 2019 (COVID-19) posed a serious global public health threat. The first prevalence of the virus was reported in Wuhan China, on 31st December 2019. According to Johns Hopkins University Coronavirus Resource Center live update (Johns Hopkins University Medicine, Coronavirus Resource Center), the total cases of COVID-19 has reached more than 144 million and caused over 3 million deaths over the globe (January 2021). The COVID-19 pandemic has been studied from diverse perspectives, and health care professionals are trying their best to control the pandemic. As the consequences of COVID-19 are potentially severe, avoiding infection with severe acute respiratory syndrome coronavirus 2 (SARS-CoV-2) is important. Certain gene expressions are found to be closely related to coronavirus infection. Since SNP frequency and existence vary among population groups, groups with gene expression-related SNPs may show higher vulnerability to COVID-19 infection. Thus, ethnicity could be suggested as a possible factor affecting the severity of SARS-CoV-2 infections [3]. The virus first emerged in East Asia, but relatively higher rates of morbidity and mortality have been identified in European populations. It is therefore important to determine the mechanism underlying the effect of ethnicity on the severity of COVID-19.

Concerning the biochemistry of SARS-CoV-2, the binding of the viral spike (S) protein to cellular receptors and priming of the S protein by host cell proteases are significant factors affecting the entry of SARS-CoV-2 into the host cell [1, 2]. Several studies have found that angiotensin-converting enzyme 2 (*ACE2*) and transmembrane protease serine 2 (*TMPRSS2*) are involved in this step. *ACE2* is the cellular receptor to which SARS-CoV-2 binds, thereby gaining entry into the host cell. *ACE2* is involved in regulatory processes in our body. *ACE2* is also the functional receptor for severe acute respiratory syndrome coronavirus (SARS-CoV). As the expression levels of *ACE2* are high in the heart and lungs, COVID-19 patients can develop heart- and lung-related complications. *TMPRSS2* cleaves the spike protein of SARS-CoV-2, leading to the activation of the virus and cellular membrane [3]. Given the involvement of these proteins in the entry of SARS-CoV-2 into host cells, the relationship between ethnicity and disease severity may be due to single-nucleotide polymorphisms (SNPs) in the corresponding genes. SNPs provide information on individuals’ possible responses to certain treatments or drugs and susceptibility to environmental elements. Thus, a systematic review on SNPs that affect susceptibility and severity of SARS-CoV-2 may enhance the individualized coronavirus treatment. Personalized drugs and treatments used to infect patients allow faster recovery by removal of insignificant processes. Also, it would minimize and prevent the side effects of certain treatments to certain patients. Therefore, in this systematic review, we aimed to discover the related SNPs of SARS-CoV-2 infection by going through all the SNPs mentioned in multiple papers on the identical topic. We analyzed papers on SNPs in the genes encoding mainly *ACE2* and *TMPRSS2* and their connections with COVID-19.

## Method

### Literature search method

PubMed was searched for relevant articles. Going through the selected number of journals related to the topic, keywords were extracted from the journals, and a Medline search expression was created. The main text words were SARS-CoV-2, COVID-19, coronavirus disease, variant genes, whole-exome sequencing, and significant linkage disequilibrium. Then, the MeSH terms were derived from the main text words chosen. Text words were searched as [tiab], and MeSH terms were searched as [MeSH] to indicate the purpose of each word. When linking the keywords, AND and OR were used according to the needs of the selected terms and keywords. The article selection process was performed by two independent reviewers. The final searching expression used is ((SARS-CoV-2[tiab] OR COVID-19[tiab] OR “Coronavirus disease”[tiab] OR “Severe acute respiratory syndrome coronavirus 2”[tiab] OR coronavirus[tiab]) **OR** (SARS-CoV-2[Mesh] OR “Spike Glycoprotein, Coronavirus”[Mesh] OR COVID-19[Mesh] OR Betacoronavirus[Mesh] OR “Coronavirus Infections”[Mesh])) **AND** ((“Variant gene”[tiab] OR “whole-exome sequencing”[tiab] OR “allele frequency”[tiab] OR mutations[tiab] OR “protein–protein interaction”[tiab] OR “Significant linkage disequilibrium”[tiab] OR LD[tiab] OR PPI[tiab] OR Variants[tiab] OR Coding[tiab] OR Missense[tiab] OR “epigenetic modification”[tiab] OR polymorphism[tiab]) **OR** (“Molecular Docking Simulation”[Mesh] OR “Protein Interaction Domains and Motifs”[Mesh] OR “Virus Internalization”[Mesh] OR “High-Throughput Nucleotide Sequencing”[Mesh] OR “Polymorphism, Single Nucleotide*”[Mesh] OR “Real-Time Polymerase Chain Reaction”[Mesh])). The search was done in 2019 December.

### Paper selection

All the papers gained from searching using the Medline expression from above were taken as initial 2956 papers. Then, the following exclusion criteria were used to exclude the papers inappropriate for inclusion in this systematic review.

The following inclusion criteria were applied:

1. Studies that bases on human subjects’ infection of coronavirus.
2. Studies directly related to the COVID-19 crisis (Study published on or after 2019 December).
3. Studies on COVID-19 that discuss genetics or the cellular infection mechanism.
4. Studies with direct mention of COVID-19 related SNPs and genes.

The following exclusion criteria were applied:

1. Animal studies and studies with human subjects involving other coronaviruses, such as bovine coronavirus and delta coronavirus.
2. Editorial letters, case reports, technical notes, meta-analyses, reviews, and systematic reviews.
3. Studies on irrelevant topics, such as porcine diarrhea.
4. Studies on COVID-19 that did not discuss genetics or the cellular infection mechanism.

### Study selection and review process

The study selection and review process was performed by 2 of us independently. Any type of discord on the study selection and review process was resolved through discussion. The following criteria were considered when selecting and reviewing studies for systematic review: type of trial, clinical and study outcomes, study population, statistically powerful results, and topic relevance. Clinical and study outcomes refer to the genes and SNPs that are mentioned to be related to SARS-CoV-19 susceptibility. Topic relevance was primarily judged by the inclusion and exclusion criteria above. Then the papers were further selected by scheming whether the paper directly mentions COVID-19 infection related genes or SNPs.

### Quality assessment

The Newcastle Ottawa Scale (NOS) was used to assess the quality of the 21 included papers. This quality assessment tool was formed by a collaboration between two universities, the University of Newcastle, Australia, and the University of Ottawa, Canada. The NOS was created for the assessment of the quality of nonrandomized studies, such as case–control and cohort studies. There are three domains in the NOS: selection, comparability, and outcome [4].

Selection considers the representativeness of the exposed cohort, selection of the non-exposed cohort, ascertainment of exposure, and demonstration that the outcome of interest was not present at the start of the study. For questions 2 and 3 in the outcome section, which asks about follow-up, all papers had to be scored as “yes.” COVID-19 is a recent issue; therefore, the follow-up duration could not be as long as in studies on other topics. The comparability of the duration of follow-up between the included studies and usual studies had to be deemed acceptable. There were four assessment questions under the *selection* section. For representativeness of the exposed cohort section, *(a) truly representative*, and *(b) somewhat representative* were both given one star. For the selection of the non-exposed cohort, *(a) Drawn from the same community as the exposed cohort* was given one star. The ascertainment of the exposure, both *(a) Secure record* and *(b) Structured interview* were given a star. The last question under the selection section demonstrated that the outcome of interest was not present at the start of the study, choice of *(a) Yes*, is only given a star. The only criteria to assess the comparability, comparability of the cohorts based on the design or analysis is controlled for cofounders, both choice *(a) The study controls for age, sex, and marital status* and *(b) Study controls for other factors* were given a star. Under the outcome section, there were three questions to follow to assess the corresponding criteria. For assessment of outcome, both choices *(a) Independent blind assessment* and *(b) Record linkage* was given a star. The second question under the outcome section was follow-up long enough for outcomes to occur, choice *(a) Yes* is given a star. The last question for assessing outcome, adequacy of follow-up of cohorts, both answer of *(a) Complete follow-up all subject accounted for* and *(b) Subjects lost to follow up unlikely to introduce bias-number lost less than or equal to 20% or description of those lost suggested no different from those followed* are the choices given a star [5].

## Results

**Fig. 1:**
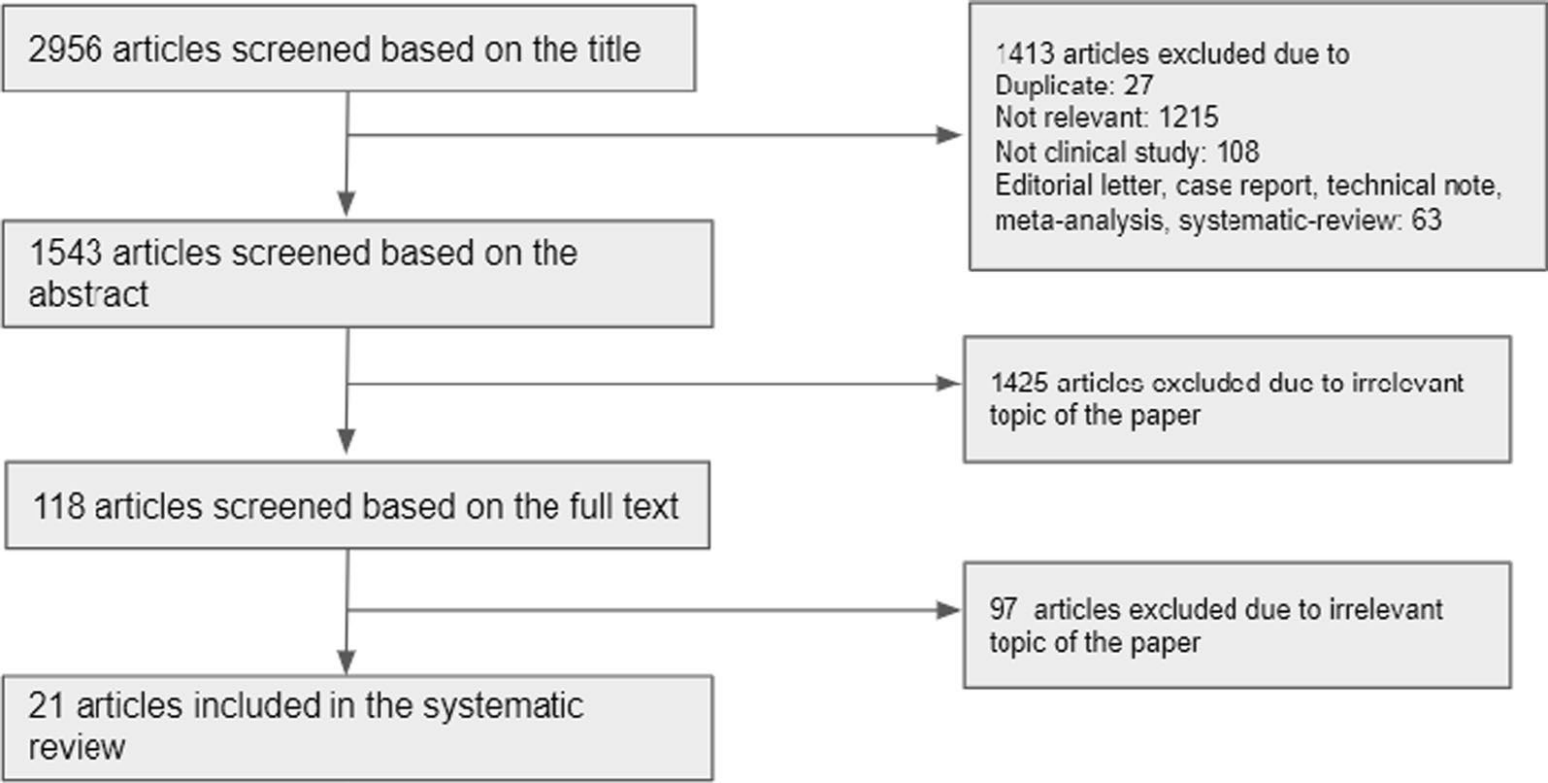
Flow chart depicting literature search and selection process

Out of 2956 papers searched initially, 21 academic papers were selected for the systematic review (Fig. 1). 21 papers discussing and analyzing genetic factors related to infection with SARS-CoV-2 were reviewed. 18 of them were published in 2020, and 3 were published in 2021. Out of the 21 papers, 5 papers [6–10] recruited patients from specific regions or hospitals. The other 16 papers used large databases, such as the 1000 Genomes Project, gnomAD, National Center for Biotechnology Information (NCBI), Global Initiative on Sharing Avian Flu Data (GISAID) Illumina, and the World Health Organization (WHO) dashboard. All papers reviewed were assessed with Newcastle Ottawa Scale and scored 8 out of 8 equivalently. In total, the mean ± standard deviation number of patients per paper was 63,496 ± 13,889.90; the numbers of patients in the databases mentioned above were not taken into account. The average age of the subjects was 55 years old; however, this value is not accurate because multiple studies did not report age or only recorded the age range and not the average age. The genes investigated in these papers were mainly *ACE2* and *TMPRSS2. IFITM3, CD147, IFIH1, IL6, LZTFL1,* and *ACE1* were also mentioned in some papers (Table 1).

**Table 1:** Characteristics of the included studies

First author (last name)	Publication year	Country	Journal	Database name	Number of subjects	Age (years) Mean (if the mean value is not mentioned in the paper, age range mentioned is recorded instead)	Gene	SNP
Maiti et al. [25]	2020	USA	Immunogenetics	1000 genome project (https://www.internationalgenome.org/)	N/A	N/A	IFIH1	rs1990760
Torre-Fuentes et al. [6]	2021	Spain	Journal of Medical Virology	MS family cohort	120	N/A	ACE2, TMPRSS2	rs75603675, rs61735792, rs61735794
Gomez et al. [7]	2021	Spain	Cytokine	Hospital Univ. Central Asturias, Spain	311	65.23	IFITM3	rs12252 (C)
Zhang et al. [8]	2020	China	The Journal of Infectious Diseases	Patients were recruited from Beijing Youan Hospital, Capital Medical University, Beijing, between January 2020 and February 2020	80	49.5	IFITM3	rs12252 (C)
Hussain et al. [11]	2020	Pakistan	Journal of Medical Virology	Ensembl (https://asia.ensembl.org/index.html), Genome Browser12 (https://genome.ucsc.edu/), gnomAD (https://gnomad.broadinstitute.org/)	N/A	N/A	ACE2	rs7363582, rs143936283
Gomez et al. [9]	2020	Spain	Elsevier	Hospital Univ. Central Asturias, Spain	740	67.44	ACE, ACE2	rs2285666
Wang et al. [12]	2020	China	Journal of General Virology	dbSNP (https://www.ncbi.nlm.nih.gov/snp/?cmd=search), National Genomics Data Center (https://ngdc.cncb.ac.cn/)	N/A	N/A	ACE2	rs143936283 rs267606406 rs4646116
Fujikura et al. [15]	2020	Japan	Journal of clinical pathology	1000G (https://www.internationalgenome.org/), NHLBI (https://www.nhlbi.nih.gov/), gnomAD, ToMMo (https://www.megabank.tohoku.ac.jp/english/), UK10K (https://www.uk10k.org/)	669	N/A	ACE2, TMPRSS2	N/A
Yamamoto et al. [16]	2020	Japan	Elsevier	High-coverage sequenced data of the phase 3 panel of the international 1000 Genomes Project (1000Genomes) and the Korean Personal Genome Project (KPGP)	N/A	N/A	ACE, ACE2	N/A
Sienko et al. [17]	2020	Poland	Clinical Interventions in Aging	N/A	6272	N/A	ACE2, TMPRSS2, CD147	N/A
Paniri et al. [18]	2021	Iran	Gene Rep	NCBI, UniProtKB (https://www.uniprot.org/), PANTHER (http://www.pantherdb.org/)	52,456	N/A	ACE2	rs149039346, rs147311723, rs714205, rs1514283, rs4646175, rs3746444, rs113808830, rs3751304
Nguyen et al. [10]	2020	Vietnam	PLoS One	A hospital in Vietnam	44	15–74	ACE2	N/A
Senapati et al. [19]	2020	India	J Ganet	GTEx (https://gtexportal.org/home/), Uniprot	26	60 or more	ACE2, TMPRSS2, CD26	rs112657409, rs11910678, rs77675406, rs713400, rs13015258
Novelli et al. [20]	2020	Italy	Human Genomics	GnomAD	131	N/A	ACE2	N/A
Vargas-Alarcón et al. [21]	2020	Mexico	ELSEVIER	dbSNPs, Ensembl Genome Browser, and 1000 Genome Project databases	N/A	N/A	ACE2, TMPRSS2, TMPRSS11A, ELANE, CTSL	rs12329760
Benetti et al. [22]	2020	Italy	European Journal of Human Genetics	NIG-db, LOVD (https://www.lovd.nl/), gnomAD,	389	N/A	ACE2	rs775181355, rs762890235
Strafella et al. [13]	2020	Italy	MDPI	Ensembl, 1000 Genomes, GnomAD	268	46	ACE2	rs35803318, rs41303171, rs774469453, rs773676270, rs2285666
Shikov et al. [23]	2020	Russia	Front Genet	gnomAD	58	N/A	ACE2	rs146598386, rs73195521, rs755766792
Srivastava et al. [14]	2020	India	Front. Genet	1000 genome project	N/A	N/A	ACE2	rs2285666
Yang et al. [26]	2020	Taiwan	PNAS	GISAID, Illumina	1932	N/A	N/A	N/A
Kim et al. [24]	2020	South Korea	MDPI	World Health Organization (WHO) COVID-19 dashboard	N/A	N/A	IFITM3, ACE2, TMPRSS2, IL6, LZTFL1	rs6598045, rs12252, rs34481144, rs6598045ss

### Quality assessment

All papers had equal quality assessment scores (Table 2). The papers varied in terms of the representativeness of the cohort. Specifically, the papers that used databases were categorized as “truly representative.” The remaining papers, which were classified as “somewhat representative,” collected genomic data from patients from a single hospital or region. Since the aim of this systematic review was to identify SNPs associated with infection with SARS-CoV-2 and the severity of COVID-19 regardless of other health factors, papers that were relevant to the purpose of the review were mostly assessed as being appropriate.

**Table 2:** Newcastle–Ottawa Scale to assess quality of studies involved in systematic review

	Selection					Outcome			
	Representativeness of expressed cohort	Selection of non expressed cohort	Ascertainment of exposure	Outcome not present at the start of the study	Comparability	Assessment of outcomes	Length of follow-up	Adequacy of follow-up	Total score
Maiti et al. [25]	a	a	a	a	a	a	a	a	8
Torre-Fuentes et al. [6]	b	a	a	a	a	a	a	a	8
Gomez et al. [7]	b	a	a	a	a	a	a	a	8
Zhang et al. [8]	b	a	a	a	a	a	a	a	8
Hussain et al. [11]	b	a	a	a	a	a	a	a	8
Gomez et al. [9]	b	a	a	a	a	a	a	a	8
Wang et al. [12]	a	a	a	a	a	a	a	a	8
Fujikura et al. [15]	a	a	a	a	a	a	a	a	8
Yamamoto et al. [16]	b	a	a	a	a	a	a	a	8
Sienko et al. [17]	a	a	a	a	a	a	a	a	8
Paniri et al. [18]	a	a	a	a	a	a	a	a	8
Nguyen et al. [10]	b	a	a	a	a	a	a	a	8
Senapati et al. [19]	b	a	a	a	a	a	a	a	8
Novelli et al. [20]	b	a	a	a	a	a	a	a	8
Vargas-Alarcón et al. [21]	b	a	a	a	a	a	a	a	8
Benetti et al. [22]	b	a	a	a	a	a	a	a	8
Strafella et al. [13]	b	a	a	a	a	a	a	a	8
Shikov et al. [23]	b	a	a	a	a	a	a	a	8
Srivastava et al. [14]	b	a	a	a	a	a	a	a	8
Yang et al. [26]	b	a	a	a	a	a	a	a	8
Kim et al. [24]	a	a	a	a	a	a	a	a	8

### Genes and SNPs related

In the 21 included papers, *ACE2* was mentioned most frequently, and *TMPRSS2* and *IFITM3* were also mentioned in some papers. Overall, there were some SNPs reported in multiple studies as being related to infection with SARS-CoV-2 and the severity of COVID-19.

As the study was performed with limited data sources and the diversity of the study populations varied, it was difficult to identify common SNPs. However, several common SNPs were found in the studies, namely rs12252-C [7, 8], rs143936283 [11, 12], rs2285666 [13, 14], rs41303171 [6, 13], and rs35803318 [6, 13] (Table 3). Two studies mentioned rs12252-C. These studies investigated *IFITM3* (transmembrane protein 3), which is known to be associated with the infection of influenza and other viral infections. Gomez et al. [7] database was on Spanish population and Zhang et al. [8] study was based on Chinese population. The rs12252 C variant is known to be highly associated with Chinese population’s influenza infection. However, as it is commonly found as a risk factor in Spanish database study suggests that rs12252 C affects all population’s SARS-CoV-2 infection including European population. The other SNPs that were investigated in multiple studies, namely, rs143936283, rs2285666, rs41303171, and rs35803318 are in *ACE2.* The papers these SNPs were measured based their study on general databases such as Ensembl, 1000 Genomes, and GnomAD. Therefore, these SNPs can’t be specified or analyzed in affecting a certain ethnic group. Moreover, when looking at the genes and corresponding related SNPs mentioned, *ACE2* and *TMPRSS2* are often indicated together. Some studies suggest that *ACE2* and *TMPRSS2* have synergistic effects together, activating the *ACE2* as an entry receptor (Table 1). In detail, *TMPRSS2* cleaves the viral spike glycoprotein (S) and leads to viral activation facilitation [3]. Adding on to the above-mentioned SNPs, rs75603675, rs2285666, rs879922, rs73635825, rs143936283, rs143936283 rs267606406 rs4646116, rs149039346, rs147311723, rs714205, rs1514283, rs4646175, rs3746444, rs113808830, rs3751304, rs112657409, rs11910678, rs77675406, rs713400, rs13015258, rs12329760, rs775181355, rs762890235, rs35803318, rs41303171, rs774469453, rs773676270, rs2285666, rs146598386, rs73195521, rs755766792, rs2285666, and rs6598045, in total 34 SNPs, showed relation with ACE2 gene action. 9 SNPs, rs61735794, rs61735792, rs75603675, rs112657409, rs11910678, rs77675406, rs713400, rs13015258, and rs12329760, were the SNPs all showed to have linkage with *TMPRSS2. IFITM3* had 2 associated SNPs mentioned out of the studies reviewed, which were rs12252-C and rs6598045 (Table 4). ACE2 had the greatest number of related SNPs and *IFITM3*, then *TMPRSS2.*

**Table 3:** SNPs mentioned twice or more in the reviewed studies

SNP	Gene	Mentioned paper
rs12252-C	IFITM3	Gomez et al. [7]
		Zhang et al. [8]
		Kim et al. [24]
rs143936283	ACE2	Hussain et al. [11]
		Wang et al. [12]
rs2285666	ACE2	Strafella et al. [13]
		Srivastava et al. [14]
rs41303171	ACE2	Torre-Fuentes et al. [6]
		Strafella et al. [13]
rs35803318	ACE2	Torre-Fuentes et al. [6]
		Strafella et al. [13]

**Table 4:** Genes mentioned twice or more in the reviewed studies and the according related SNPs mentioned

Gene	Related SNPs	Role of the gene
ACE2 (angiotensin I converting enzyme 2)	rs75603675, rs2285666, rs879922, rs73635825, rs143936283, rs143936283 rs267606406 rs4646116, rs149039346, rs147311723, rs714205, rs1514283, rs4646175, rs3746444, rs113808830, rs3751304, rs112657409, rs11910678, rs77675406, rs713400, rs13015258, rs12329760, rs775181355, rs762890235, rs35803318, rs41303171, rs774469453, rs773676270, rs2285666, rs146598386, rs73195521, rs755766792, rs2285666, rs6598045	SARS-CoV-2 spike protein entry receptor [15]
IFITM3 (interferon-induced transmembrane protein 3)	rs12252-C, rs6598045	Gene variants of IFITM3 are related to the infection of influenza and viruses. IFITM3 is significant in taking antiviral actions. It prevents cellular lipid bilayer from getting bisected by viruses [7]. Immune effector protein that is significant to the restriction of a virus is encoded by IFITM3. Also, membrane restriction is done by IFITM3 [8]
TMPRSS2 (transmembrane protease, serine 2)	rs61735794, rs61735792, rs75603675, rs112657409, rs11910678, rs77675406, rs713400, rs13015258, rs12329760	Cleavage of TMPRSS2 activates influenza virus hemagglutinin and the human metapneumovirus F protein [3]

### SNP related to disease severity

There were only a few studies that discusses COVID-19 disease severity in depth. 3 papers mentioned the rs12252 that is found in severe COVID-19 patients [7, 8, 24]. In the studies, after classifying the patients according to their degree of the disease, rs12252 in the IFITM3 gene was commonly observed in severe COVID-19 patients. Analyzing the genotype of rs12252, the C carrier of rs12252 in the IFITM3 gene was found to be most frequent. 12 out of 61 individuals (19.7%) with rs12252 C carrier (CT + CC) were classified as a severe patient group. 69 out of 690 individuals (10%) with rs12252 TT homozygote were classified as a severe patient group. Even calculating the ratio of severe patients excluding the control group, C carriers showed greater value. According to the cohort study by Gomez et al., there were only 3 out of 751 individuals in the cohort with rs12252 CC homozygote. All three of them were COVID-19 infected patients and 2 of them were in the severe group [7]. In the study by Zhang et al., patients with CC + CT homozygotes and TT homozygotes showed a similar ratio of severe patients. However, a total of three individuals that died of the disease were all rs12252 C carriers [8]. Overall, rs12252 is possibly a COVID-19 risk developing related genetic factor. More specifically, individuals with rs12252 C carrier may have a greater likelihood of developing severe COVID-19 disease.

## Discussion

This study is started from interest and curiosity on the studies that suggest a certain group of people has greater susceptibility to SARS-CoV-2. Therefore, the study aims to find out genes and SNPs that are related to SARS-CoV-2 infection severity. 21 papers are in-depth reviewed to analyze the highly associated or frequently mentioned genetic factors. We can conclude that genetic susceptibility to infection with SARS-CoV-2 mainly involves *ACE2* and *TMPRSS2*. Torre-Fuentes et al. [6], Hussain et al. [11], Gomez et al. [9], Wang et al. [12], Fujikura et al. [15], Yamamoto et al. [16], Sienko et al. [17], Paniri et al. [18], Nguyen et al. [10], Senapati et al. [19], Novelli et al. [20], Vargas-Alarcon et al. [21], Benetti et al. [22], Strafella et al. [13], Shikov et al. [23], Srivastava et al. [14], and Kim et al. [24] mention *ACE2* as SARS-CoV-2 susceptibility related gene. Fujikura et al. [15], Sienko et al. [17], Senapati et al. [19], Vargas-Alarcon et al. [21], and Kim et al. [24] mention *TMPRSS2*. Gomez et al. [7], Zhang et al. [8], and Kim et al. [24] discuss *IFITM3* as genes that are associated with coronavirus 2019 infection severity.

As the severity and SNP have not been stated with enough statistical significance, “Results" and "Discussion” sections of each study were referred for discussing relevance between SNPs and disease severity. Ethnicity, age, sex, and other disease infection relevance were mentioned as severity-associated factors. The upregulation of *TMPRSS2* gene expression is found to be more commonly found in Italians when compared to the East Asian population [3, 27]. rs12252, related to the *IFITM3* gene, shows some relevance with the Han Chinese population’s COVID-19 severity [24]. This is due to rs12252 C/T being linked with the avian influenza virus severity and with grievous illness in influenza H1N1/09 virus-infected Chinese Han adults [7]. As large cohorts mostly come from Chinese Hospitals, Chinese populations were mentioned most frequently and studied widely. However, this does not necessarily mean the Chinese population shows greater disease severity compared with other ethnicities. Furthermore, referring to Gomez et al. [9], hypertension patients and males are referred to as independent significant predictors of severity. Zhang’s [8] study showed greater disease severity in individuals aged greater than 63.5-year-old.

This study is the first study to gather the genetic studies related to SARS-CoV-2 infection and suggest an analyzed tendency of the data. As COVID-19 is the issue of greatest attention, and there is no determinate cure for the infection of the virus, this study may somehow suggest ways for research in SARS-CoV-2 infection cures. Looking at most of the papers that deal with genetic factors of SARS-CoV-2 infection severity, clearly tells which factors should be focused on and targeted. Also, by further studies of the genes and SNPs mentioned, which biological characteristics of people are comparably more vulnerable to the disease infection. Knowing which groups are more vulnerable and what traits make infection or disease development, prevention of epidemics may be improved.

COVID-19 has only been studied for approximately 1 year; therefore, there are limitations regarding identifying genetic factors related to susceptibility to infection with SARS-CoV-2. First, genetic factors affecting susceptibility to infection and severity of disease have not yet been investigated separately. Some studies have suggested that, unlike Asians, Caucasians have SNPs that make them more susceptible to severe COVID-19. In one study, out of 5 ethnicities studied (African, European, American, East Asian, South Asian), Europeans showed the highest fatality rate of 7.4% [24]. The papers did not clearly state which SNPs are involved in infection with SARS-CoV-2 and which are involved in disease progression. It would be useful to investigate these two topics separately in future studies.

Second, the strength of the effects of these SNPs on susceptibility to infection with SARS-CoV-2 should be quantified in future studies. As progression disease is affected by the health status of the individual patient, the strength of the contribution of genetics could be challenging to quantify. However, the genes and SNPs could be ordered in terms of their relative contributions. Third, the SNPs that were mentioned to be related to certain gene’s actions (Tables 3 and 4), do not straight away affect the mechanism. Several SNPs affect the genes’ action via indirect path; therefore, it may not always apply to all individuals. As they are from all different studies that had different approaches, the SNPs collected may not necessarily affect COVID-19 infection. Lastly, as the COVID-19 pandemic developed recently and is ongoing, there were limitations to performing a systematic review. The papers included in this review were mainly published in 2020, with a few published in 2021. As the pandemic started in late 2019, papers on the topic were limited to 2019–2021. This limited the duration of clinical follow-up. Therefore, in the outcome section of the quality assessment of the articles, the second criterion, which pertains to follow-up, had to be marked as “yes.” Normally, clinical follow-up of less than 1 year would not be assessed as “yes.” In this case, long-term follow-up was impossible due to the recent cause of the pandemic.

## Conclusion

*ACE2, TMPRSS2,* and *IFITM3* were found to be the most frequently mentioned genes that are associated with SARS-CoV-2 infection. 5 SNPs were found common in two or more studies (rs12252-C, rs143936283, rs2285666, rs41303171, and rs35803318). Especially, rs12252 C carrier was mentioned in three papers in common as SNP found in severe COVID-19 patients. These SNPs are all related to the genes mentioned above. Although there were some limitations due to lack of data range and follow-up time, this study still suggests a general genetic characteristic of vulnerable SARS-CoV-2 infection. Future further research may be done to specify the exact impact of the SNP in terms of severity and degree of impact.

## Data Availability

The studies included in the systematic review are all retrieved from PubMed.gov. All studies included can be found at PubMed.gov. List and url links of Databases mentioned in the Studies from PubMed.gov: 1000 genome project (https://www.internationalgenome.org/). Ensembl (https://asia.ensembl.org/index.html). Genome Browser12 (https://genome.ucsc.edu/). gnomAD (https://gnomad.broadinstitute.org/). dbSNP (https://www.ncbi.nlm.nih.gov/snp/?cmd=search). National Genomics Data Center (https://ngdc.cncb.ac.cn/). 1000G (https://www.internationalgenome.org/). NHLBI (https://www.nhlbi.nih.gov/). ToMMo (https://www.megabank.tohoku.ac.jp/english/). UK10K (https://www.uk10k.org/). UniProtKB (https://www.uniprot.org/). PANTHER (http://www.pantherdb.org/). GTEx (https://gtexportal.org/home/). LOVD (https://www.lovd.nl/).
